# 2-Benzoyl-4-chloro­aniline thio­semi­carbazone

**DOI:** 10.1107/S1600536814011027

**Published:** 2014-05-17

**Authors:** Katlen C. T. Bandeira, Leandro Bresolin, Ueslei Z. Lehmann, Priscilla J. Zambiazi, Adriano Bof de Oliveira

**Affiliations:** a Escola de Química e Alimentos, Universidade Federal do Rio Grande, Av. Itália km 08, Campus Carreiros, 96203-900, Rio Grande-RS, Brazil; b Departamento de Química, Universidade Federal de Santa Maria, Av. Roraima, Campus, 97105-900, Santa Maria-RS, Brazil; c Departamento de Química, Universidade Federal de Sergipe, Av. Marechal Rondon s/n, Campus, 49100-000, São Cristóvão-SE, Brazil

## Abstract

In the title compound, C_14_H_13_ClN_4_S, obtained from a reaction of 2-benzoyl-4-chloro­aniline with thio­semicarbazide in ethanol, the dihedral angle between the aromatic rings is 81.31 (13)°. In the crystal, the mol­ecules are linked by three N—H⋯S hydrogen bonds, forming centrosymmetric rings with set-graph motif *R*_2_^2^(8) and *R*_2_^2^(18), and resulting in the formation of a two-dimensional network lying parallel to (010).

## Related literature   

For the coordination chemistry of thio­semicarbazone compounds, see: Lobana *et al.* (2009 ▶). For one of the first reports of the synthesis of a thio­semicarbazone derivative, see: Freund & Schander (1902 ▶). For hydrogen-bond motifs, see: Bernstein *et al.* (1995 ▶).
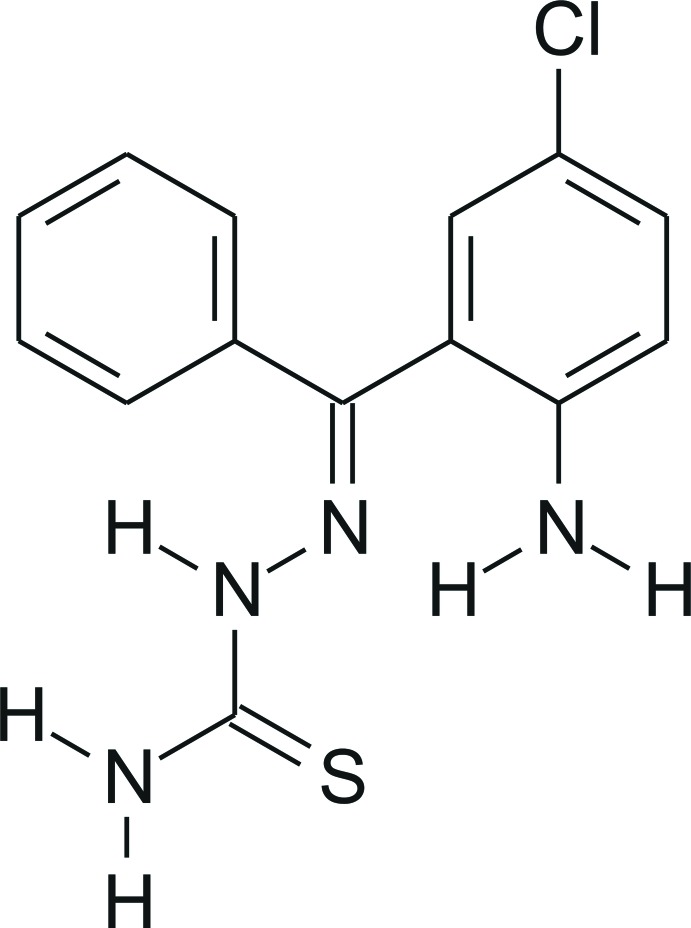


## Experimental   

### 

#### Crystal data  

C_14_H_13_ClN_4_S
*M* *_r_* = 304.79
Monoclinic, 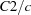

*a* = 22.46 (5) Å
*b* = 6.773 (14) Å
*c* = 19.28 (4) Å
β = 102.22 (6)°
*V* = 2866 (10) Å^3^
*Z* = 8
Mo *K*α radiation
μ = 0.41 mm^−1^
*T* = 298 K
1.14 × 0.31 × 0.16 mm

#### Data collection  

Bruker APEXII CCD diffractometer
Absorption correction: multi-scan (*SADABS*; Bruker, 2009 ▶) *T*_min_ = 0.654, *T*_max_ = 0.937
40582 measured reflections
4016 independent reflections
3348 reflections with *I* > 2σ(*I*)
*R*_int_ = 0.049

#### Refinement  

*R*[*F* ^2^ > 2σ(*F* ^2^)] = 0.044
*wR*(*F* ^2^) = 0.115
*S* = 1.10
4016 reflections
199 parameters
H atoms treated by a mixture of independent and constrained refinement
Δρ_max_ = 0.54 e Å^−3^
Δρ_min_ = −0.34 e Å^−3^

### 

Data collection: *APEX2* (Bruker, 2009 ▶); cell refinement: *SAINT* (Bruker, 2009 ▶); data reduction: *SAINT*; program(s) used to solve structure: *SHELXS97* (Sheldrick, 2008 ▶); program(s) used to refine structure: *SHELXL97* (Sheldrick, 2008 ▶); molecular graphics: *DIAMOND* (Brandenburg, 2006 ▶); software used to prepare material for publication: *publCIF* (Westrip, 2010 ▶).

## Supplementary Material

Crystal structure: contains datablock(s) I. DOI: 10.1107/S1600536814011027/bx2459sup1.cif

Structure factors: contains datablock(s) I. DOI: 10.1107/S1600536814011027/bx2459Isup2.hkl

Supporting information file. DOI: 10.1107/S1600536814011027/bx2459Isup3.cml

CCDC reference: 1002774

Additional supporting information:  crystallographic information; 3D view; checkCIF report

## Figures and Tables

**Table 1:** Hydrogen-bond geometry (Å, °)

*D*—H⋯*A*	*D*—H	H⋯*A*	*D*⋯*A*	*D*—H⋯*A*
N4—H1⋯S1^i^	0.87 (3)	2.75 (3)	3.534 (6)	150 (2)
N4—H2⋯S1^ii^	0.86 (3)	2.62 (3)	3.438 (5)	160 (2)
N3—H3*A*⋯S1^iii^	0.88 (3)	2.74 (3)	3.552 (5)	154 (2)

Symmetry codes: (i) 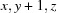; (ii) 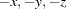; (iii) 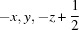.

**Fig. 1.**
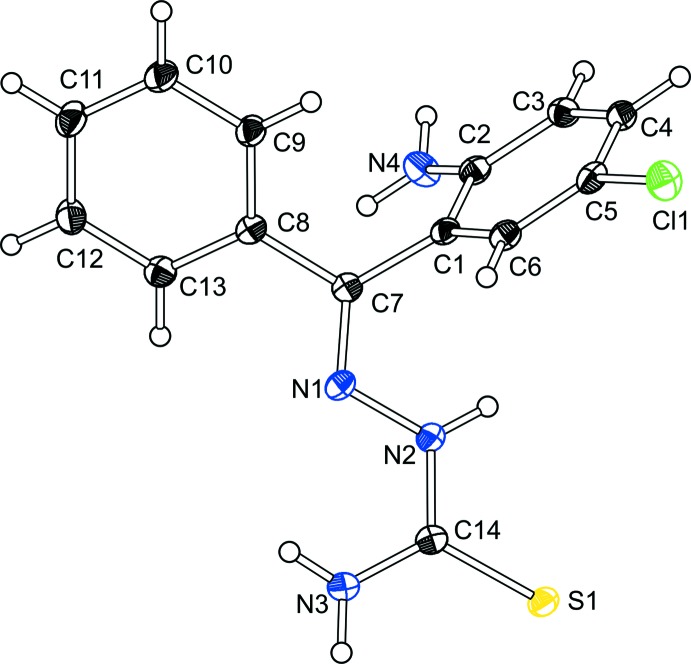
The molecular structure of the title compound with labeling and displacement ellipsoids drawn at the 50% probability level.

**Fig. 2.**
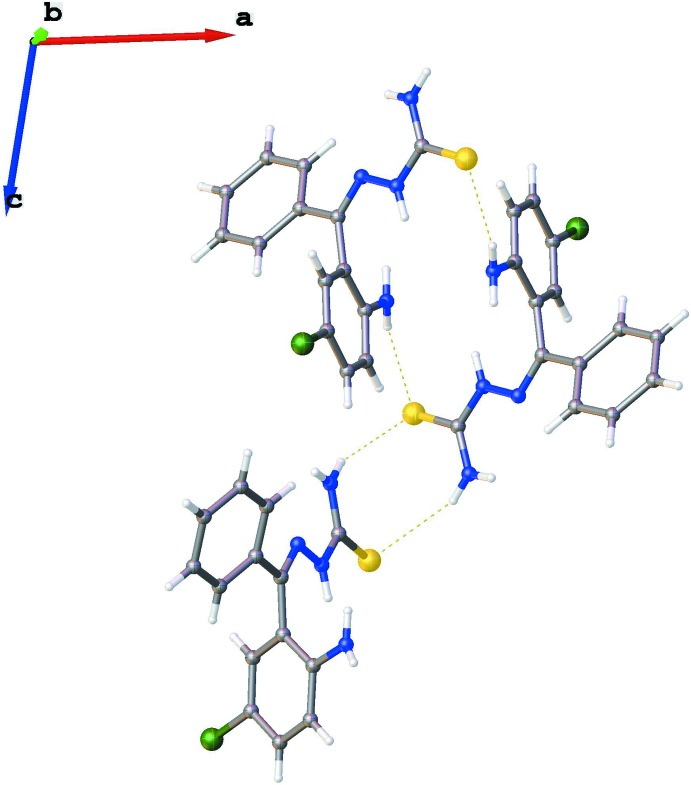
Part of the crystal structure of the title compound with view along the b-axis. The hydrogen interactions are shown as dashed lines.

## supplementary crystallographic information

### 1. Comment

Thiosemicarbazone derivatives are well know as *N*,*S*-donors with a wide range of coordination modes (Lobana *et al.*, 2009). As part of our interest on the coordination chemistry of thiosemicarbazone ligands, we report herein the synthesis and the crystal structure of a new ligand with *N*- and *Cl*-donor atoms.

The title compound (Fig. 1) is not planar and the dihedral angle between the two aromatic rings amount to 81.31 (13)°. The thiosemicarbazone fragment is almost planar, showing the torsion angle of 178.37 (12)° for the N1/N2/C14/S1 atoms. Additionally, the molecule shows a *trans* conformation for the atoms about the N1—N2 bond.

The mean deviations from the least squares plane for the aromatic ring with *-NH_2_* and *-Cl* fragments amount to 0.0371 (12) Å for N4 which implies on a planar geommetry. The *N*- and *Cl*-donor atoms can increase the number of coordination modes and the dimensionality of the coordination polymers.

In the title compound, C_14_H_13_ClN_4_S, the molecule is not planar, the dihedral angle between the two aromatic rings amount to 81.31 (13)°. In the crystal structure the molecules are linked by three N—H···S hydrogen bonds (Table 1) interactions forming centrosymmetric rings with set-graph motif R_2_^2^ (8) and R_2_^2^ (18) (Bernstein, *et al.*, 1995) and resulting in the formation of a two-dimensional network lying parallel to (010), Fig. 2. (Dolomanov *et al.*, 2009).

### 2. Experimental

Starting materials were commercially available and were used without further purification. The synthesis was adapted from a procedure reported previously (Freund & Schander, 1902). The hydrochloric acid catalyzed reaction of 4-chloro-2-benzoylaniline (8,83 mmol) and thiosemicarbazide (8,83 mmol) in ethanol (50 ml) was refluxed for 6 h. After cooling and filtering, the title compound was obtained. Crystals suitable for X-ray diffraction of 4-chloro-2-benzoylaniline thiosemicarbazone were obtained in ethanol by the slow evaporation of the solvent.

### 3. Refinement

All aromatic H atoms were positioned with idealized geometry and were refined isotropic with *U*_iso_(H) = 1.2 *U*_eq_ using a riding model with C—H = 0.93 Å. The amine and hydrazine H atoms were located in difference map but were positioned with idealized geometry and refined isotropic with *U*_iso_(H) = 1.2 *U*_eq_ using a riding model with N2—H2A = 0.87 (2), N3—H3A = 0.88 (3), N3—H3B = 0.85 (3), N4—H1 = 0.87 (3) and N4—H2 = 0.86 (3) Å.

### Crystal data

**Table:**

C_14_H_13_ClN_4_S	*F*(000) = 1264
*M**_r_* = 304.79	*D*_x_ = 1.413 Mg m^−^^3^
Monoclinic, *C*2/*c*	Mo *K*α radiation, λ = 0.71073 Å
Hall symbol: -C 2yc	Cell parameters from 4016 reflections
*a* = 22.46 (5) Å	θ = 2.2–29.8°
*b* = 6.773 (14) Å	µ = 0.41 mm^−^^1^
*c* = 19.28 (4) Å	*T* = 298 K
β = 102.22 (6)°	Needle, yellow
*V* = 2866 (10) Å^3^	1.14 × 0.31 × 0.16 mm
*Z* = 8	

### Data collection

**Table:**

Bruker APEXII CCD diffractometer	4016 independent reflections
Radiation source: fine-focus sealed tube, Bruker APEX-II CCD	3348 reflections with *I* > 2σ(*I*)
Graphite monochromator	*R*_int_ = 0.049
φ and ω scans	θ_max_ = 29.8°, θ_min_ = 2.2°
Absorption correction: multi-scan (*SADABS*; Bruker, 2009)	*h* = −30→30
*T*_min_ = 0.654, *T*_max_ = 0.937	*k* = −9→9
40582 measured reflections	*l* = −26→26

### Refinement

**Table:**

Refinement on *F*^2^	Primary atom site location: structure-invariant direct methods
Least-squares matrix: full	Secondary atom site location: difference Fourier map
*R*[*F*^2^ > 2σ(*F*^2^)] = 0.044	Hydrogen site location: inferred from neighbouring sites
*wR*(*F*^2^) = 0.115	H atoms treated by a mixture of independent and constrained refinement
*S* = 1.10	*w* = 1/[σ^2^(*F*_o_^2^) + (0.0481*P*)^2^ + 5.8502*P*] where *P* = (*F*_o_^2^ + 2*F*_c_^2^)/3
4016 reflections	(Δ/σ)_max_ < 0.001
199 parameters	Δρ_max_ = 0.54 e Å^−^^3^
0 restraints	Δρ_min_ = −0.34 e Å^−^^3^

### Special details

**Table:**

Geometry. All e.s.d.'s (except the e.s.d. in the dihedral angle between two l.s. planes) are estimated using the full covariance matrix. The cell e.s.d.'s are taken into account individually in the estimation of e.s.d.'s in distances, angles and torsion angles; correlations between e.s.d.'s in cell parameters are only used when they are defined by crystal symmetry. An approximate (isotropic) treatment of cell e.s.d.'s is used for estimating e.s.d.'s involving l.s. planes.
Refinement. Refinement of *F*^2^ against ALL reflections. The weighted *R*-factor *wR* and goodness of fit *S* are based on *F*^2^, conventional *R*-factors *R* are based on *F*, with *F* set to zero for negative *F*^2^. The threshold expression of *F*^2^ > σ(*F*^2^) is used only for calculating *R*-factors(gt) *etc*. and is not relevant to the choice of reflections for refinement. *R*-factors based on *F*^2^ are statistically about twice as large as those based on *F*, and *R*- factors based on ALL data will be even larger.

### Fractional atomic coordinates and isotropic or equivalent isotropic displacement parameters (Å^2^)

**Table:**

	*x*	*y*	*z*	*U*_iso_*/*U*_eq_	
Cl1	0.16609 (2)	−0.46293 (7)	−0.09583 (3)	0.02355 (12)	
S1	0.01214 (2)	−0.54784 (6)	0.14910 (2)	0.01662 (11)	
N1	0.11537 (7)	−0.0755 (2)	0.15219 (8)	0.0157 (3)	
N2	0.07789 (7)	−0.2359 (2)	0.12998 (8)	0.0170 (3)	
N3	0.08055 (7)	−0.3151 (3)	0.24678 (9)	0.0193 (3)	
N4	0.05109 (8)	0.2589 (2)	−0.00573 (9)	0.0207 (3)	
C11	0.25090 (9)	0.5202 (3)	0.16607 (11)	0.0215 (4)	
H11	0.2774	0.6251	0.1802	0.026*	
C12	0.21147 (8)	0.4588 (3)	0.20907 (10)	0.0199 (4)	
H12	0.2116	0.5238	0.2516	0.024*	
C13	0.17195 (8)	0.3005 (3)	0.18844 (10)	0.0168 (3)	
H13	0.1456	0.2606	0.2171	0.020*	
C8	0.17192 (7)	0.2011 (2)	0.12438 (9)	0.0145 (3)	
C7	0.13232 (7)	0.0255 (3)	0.10233 (9)	0.0146 (3)	
C1	0.11700 (7)	−0.0301 (3)	0.02510 (9)	0.0144 (3)	
C6	0.14226 (8)	−0.2037 (3)	0.00290 (10)	0.0164 (3)	
H6	0.1655	−0.2871	0.0364	0.020*	
C5	0.13252 (8)	−0.2503 (2)	−0.06868 (10)	0.0165 (3)	
C4	0.09716 (8)	−0.1273 (3)	−0.11973 (10)	0.0171 (3)	
H4	0.0910	−0.1587	−0.1677	0.020*	
C14	0.05977 (7)	−0.3555 (2)	0.17820 (9)	0.0147 (3)	
C3	0.07136 (8)	0.0420 (3)	−0.09813 (10)	0.0167 (3)	
H3	0.0478	0.1230	−0.1321	0.020*	
C2	0.08014 (8)	0.0942 (3)	−0.02549 (10)	0.0155 (3)	
C9	0.21096 (8)	0.2656 (3)	0.08098 (10)	0.0178 (3)	
H9	0.2105	0.2024	0.0380	0.021*	
C10	0.25047 (9)	0.4241 (3)	0.10199 (11)	0.0215 (4)	
H10	0.2765	0.4655	0.0732	0.026*	
H1	0.0563 (12)	0.290 (4)	0.0392 (15)	0.032*	
H2	0.0420 (12)	0.354 (4)	−0.0359 (14)	0.032*	
H3A	0.0697 (11)	−0.390 (4)	0.2792 (13)	0.024 (6)*	
H3B	0.1039 (12)	−0.216 (4)	0.2583 (14)	0.031 (7)*	
H2A	0.0635 (11)	−0.259 (4)	0.0853 (13)	0.023 (6)*	

### Atomic displacement parameters (Å^2^)

**Table:**

	*U*^11^	*U*^22^	*U*^33^	*U*^12^	*U*^13^	*U*^23^
Cl1	0.0263 (2)	0.0203 (2)	0.0249 (2)	0.00393 (17)	0.00743 (18)	−0.00452 (17)
S1	0.0159 (2)	0.0157 (2)	0.0191 (2)	−0.00251 (15)	0.00555 (16)	−0.00047 (15)
N1	0.0126 (6)	0.0153 (7)	0.0191 (7)	−0.0019 (5)	0.0032 (5)	0.0004 (5)
N2	0.0165 (7)	0.0182 (7)	0.0156 (7)	−0.0046 (6)	0.0021 (6)	0.0005 (6)
N3	0.0207 (8)	0.0208 (7)	0.0171 (8)	−0.0050 (6)	0.0056 (6)	0.0007 (6)
N4	0.0255 (8)	0.0197 (7)	0.0174 (8)	0.0075 (6)	0.0052 (6)	0.0016 (6)
C11	0.0165 (8)	0.0225 (9)	0.0247 (10)	−0.0053 (7)	0.0026 (7)	−0.0028 (7)
C12	0.0188 (8)	0.0222 (9)	0.0186 (9)	−0.0016 (7)	0.0037 (7)	−0.0046 (7)
C13	0.0121 (7)	0.0201 (8)	0.0183 (8)	0.0001 (6)	0.0038 (6)	0.0007 (7)
C8	0.0112 (7)	0.0141 (7)	0.0177 (8)	0.0010 (6)	0.0021 (6)	0.0018 (6)
C7	0.0111 (7)	0.0161 (7)	0.0169 (8)	0.0014 (6)	0.0033 (6)	0.0002 (6)
C1	0.0114 (7)	0.0157 (7)	0.0167 (8)	−0.0028 (6)	0.0045 (6)	−0.0001 (6)
C6	0.0139 (7)	0.0153 (8)	0.0199 (9)	−0.0004 (6)	0.0034 (6)	0.0012 (6)
C5	0.0142 (7)	0.0131 (7)	0.0232 (9)	−0.0011 (6)	0.0066 (6)	−0.0027 (6)
C4	0.0148 (7)	0.0200 (8)	0.0169 (8)	−0.0038 (6)	0.0047 (6)	−0.0012 (7)
C14	0.0116 (7)	0.0151 (7)	0.0181 (8)	0.0019 (6)	0.0049 (6)	0.0011 (6)
C3	0.0135 (7)	0.0200 (8)	0.0167 (8)	−0.0009 (6)	0.0030 (6)	0.0018 (6)
C2	0.0124 (7)	0.0156 (7)	0.0194 (8)	−0.0008 (6)	0.0057 (6)	0.0003 (6)
C9	0.0163 (8)	0.0190 (8)	0.0193 (9)	−0.0034 (6)	0.0061 (6)	−0.0029 (7)
C10	0.0185 (8)	0.0239 (9)	0.0239 (9)	−0.0068 (7)	0.0081 (7)	−0.0024 (7)

### Geometric parameters (Å, º)

**Table:**

Cl1—C5	1.756 (3)	C13—C8	1.406 (3)
S1—C14	1.704 (3)	C13—H13	0.9300
N1—C7	1.301 (3)	C8—C9	1.404 (3)
N1—N2	1.386 (3)	C8—C7	1.492 (3)
N2—C14	1.359 (3)	C7—C1	1.503 (4)
N2—H2A	0.87 (2)	C1—C6	1.411 (3)
N3—C14	1.334 (3)	C1—C2	1.415 (3)
N3—H3A	0.88 (3)	C6—C5	1.387 (4)
N3—H3B	0.85 (3)	C6—H6	0.9300
N4—C2	1.386 (3)	C5—C4	1.401 (3)
N4—H1	0.87 (3)	C4—C3	1.387 (3)
N4—H2	0.86 (3)	C4—H4	0.9300
C11—C10	1.395 (4)	C3—C2	1.417 (4)
C11—C12	1.398 (3)	C3—H3	0.9300
C11—H11	0.9300	C9—C10	1.397 (3)
C12—C13	1.395 (3)	C9—H9	0.9300
C12—H12	0.9300	C10—H10	0.9300
			
C7—N1—N2	115.96 (19)	C6—C1—C7	119.12 (16)
C14—N2—N1	120.42 (19)	C2—C1—C7	120.74 (19)
C14—N2—H2A	118.2 (16)	C5—C6—C1	120.16 (16)
N1—N2—H2A	121.3 (16)	C5—C6—H6	119.9
C14—N3—H3A	119.8 (16)	C1—C6—H6	119.9
C14—N3—H3B	119.1 (18)	C6—C5—C4	120.66 (19)
H3A—N3—H3B	121 (2)	C6—C5—Cl1	119.73 (14)
C2—N4—H1	119.6 (18)	C4—C5—Cl1	119.60 (18)
C2—N4—H2	118.1 (18)	C3—C4—C5	119.4 (2)
H1—N4—H2	117 (3)	C3—C4—H4	120.3
C10—C11—C12	120.0 (2)	C5—C4—H4	120.3
C10—C11—H11	120.0	N3—C14—N2	117.6 (2)
C12—C11—H11	120.0	N3—C14—S1	123.13 (14)
C13—C12—C11	120.3 (2)	N2—C14—S1	119.26 (18)
C13—C12—H12	119.9	C4—C3—C2	121.54 (17)
C11—C12—H12	119.9	C4—C3—H3	119.2
C12—C13—C8	119.99 (18)	C2—C3—H3	119.2
C12—C13—H13	120.0	N4—C2—C1	122.0 (2)
C8—C13—H13	120.0	N4—C2—C3	119.82 (17)
C9—C8—C13	119.38 (19)	C1—C2—C3	118.11 (19)
C9—C8—C7	119.21 (18)	C10—C9—C8	120.3 (2)
C13—C8—C7	121.40 (17)	C10—C9—H9	119.8
N1—C7—C8	117.31 (19)	C8—C9—H9	119.8
N1—C7—C1	123.96 (19)	C11—C10—C9	120.03 (19)
C8—C7—C1	118.65 (15)	C11—C10—H10	120.0
C6—C1—C2	120.1 (2)	C9—C10—H10	120.0
			
C7—N1—N2—C14	177.28 (15)	C1—C6—C5—C4	−0.6 (3)
C10—C11—C12—C13	0.5 (3)	C1—C6—C5—Cl1	178.09 (13)
C11—C12—C13—C8	0.4 (3)	C6—C5—C4—C3	−0.5 (3)
C12—C13—C8—C9	−1.4 (3)	Cl1—C5—C4—C3	−179.16 (13)
C12—C13—C8—C7	177.14 (16)	N1—N2—C14—N3	−1.6 (2)
N2—N1—C7—C8	179.05 (14)	N1—N2—C14—S1	178.37 (12)
N2—N1—C7—C1	−4.1 (2)	C5—C4—C3—C2	0.3 (3)
C9—C8—C7—N1	154.10 (17)	C6—C1—C2—N4	175.60 (16)
C13—C8—C7—N1	−24.4 (2)	C7—C1—C2—N4	−7.5 (3)
C9—C8—C7—C1	−22.9 (2)	C6—C1—C2—C3	−2.0 (2)
C13—C8—C7—C1	158.57 (17)	C7—C1—C2—C3	174.95 (15)
N1—C7—C1—C6	−66.7 (3)	C4—C3—C2—N4	−176.71 (16)
C8—C7—C1—C6	110.1 (2)	C4—C3—C2—C1	0.9 (3)
N1—C7—C1—C2	116.3 (2)	C13—C8—C9—C10	1.4 (3)
C8—C7—C1—C2	−66.8 (3)	C7—C8—C9—C10	−177.10 (16)
C2—C1—C6—C5	1.8 (3)	C12—C11—C10—C9	−0.4 (3)
C7—C1—C6—C5	−175.14 (15)	C8—C9—C10—C11	−0.5 (3)

### Hydrogen-bond geometry (Å, º)

**Table:**

*D*—H···*A*	*D*—H	H···*A*	*D*···*A*	*D*—H···*A*
N4—H1···S1^i^	0.87 (3)	2.75 (3)	3.534 (6)	150 (2)
N4—H2···S1^ii^	0.86 (3)	2.62 (3)	3.438 (5)	160 (2)
N3—H3*A*···S1^iii^	0.88 (3)	2.74 (3)	3.552 (5)	154 (2)

Symmetry codes: (i) *x*, *y*+1, *z*; (ii) −*x*, −*y*, −*z*; (iii) −*x*, *y*, −*z*+1/2.
